# Case report of hypnic headache: a rare headache disorder with nocturnal symptoms

**DOI:** 10.1186/s13104-017-2641-6

**Published:** 2017-07-25

**Authors:** Kanishka P. Dissanayake, Damindi P. Wanniarachchi, Udaya K. Ranawaka

**Affiliations:** 1 University Medical Unit, Colombo North Teaching Hospital, Ragama, Sri Lanka; 2 Colombo North Teaching Hospital, Ragama, Sri Lanka; 3 Department of Medicine, Faculty of Medicine, University of Kelaniya, Ragama, Sri Lanka

**Keywords:** Case report, Headache, Nocturnal headache, Hypnic headache

## Abstract

**Background:**

Headache is one of the commonest complaints reported to physicians worldwide. Yet, arriving at the proper diagnosis can be a challenge in many patients. Although most headaches belong to common categories of migraine and tension-type headache, which are diagnosed and managed relatively easily, several uncommon headache disorders can lead to delays in diagnosis. Certain medications are more efficacious than others in managing these headache disorders, hence establishing the correct diagnosis is of paramount importance.

**Case presentation:**

An 86-year-old female presented with chronic daily headache of 1 year duration. Her headaches were exclusively nocturnal and woke her up daily around midnight. Clinical examination was unremarkable. All basic investigations were normal. Subsequent gadolinium enhanced Magnetic Resonance Imaging (MRI) brain did not show any significant pathology. There was no satisfactory response to paracetamol, diclofenac sodium, mefenamic acid, tramadol, flunarizine and sodium valproate. Indomethacin was started with the provisional diagnosis of hypnic headache. There was absolute response by day 3 of indomethacin. She remains headache free on low dose indomethacin maintenance at 1 year after the diagnosis.

**Conclusion:**

Better understanding of uncommon headache syndromes can help in early diagnosis and appropriate treatment. Hypnic headache should be considered in the differential diagnosis of chronic daily headaches, especially when nocturnal and occurs during sleep.

## Background

Headache is one of the commonest complaints encountered by clinicians worldwide. Although a majority of headaches belong to the well-known categories such as migraine and tension type headache, there are several uncommon headache disorders that can pose diagnostic dilemmas. Not infrequently, patients are subjected to a battery of advanced investigations and multiple drug therapies before the correct diagnosis is made. Lack of awareness of these disorders can lead to delays in diagnosis even when patients present with classical features. Also, some medications are better than others in managing certain headache types and can give rapid relief.

## Case presentation

An 86-year-old female without significant past medical history, presented with headache of 1 year duration. Headache was exclusively nocturnal, occurring each night around 12 a.m., waking the patient up and lasting 2–3 h. She graded the severity of headache as ranging from 4 to 7 on a scale of 1 to 10 (10 being the most severe). It was unilateral, occurring mainly around the right orbital region, sometimes involving the auricular area and was described as a dull aching, non-pulsating headache without radiation. Sometimes the headache was associated with right sided rhinorrhea. There was no conjunctival injection, tearing, nausea, vomiting or photo-/phonophobia. She reports uninterrupted sleep prior to the onset of these headache episodes. There were no features to suggest obstructive sleep apnoea or other sleep related disorders. She did not give a past history of migraines or recurrent headaches, seizures, neuralgias or other neurological disease. Family history was unremarkable. She had multiple medical consultations with poor resolution of symptoms. On examination she appeared well at rest with a regular heart rate of 70 beats/min, blood pressure of 142/68 mmHg and clear lung fields. Ophthalmoscopy was normal and temporal arteries were pulsatile and non-tender. The neurological examination was unremarkable. Oxygen saturation was 97% on room air using pulse oximetry. Haematological and biochemical investigations including full blood count, blood picture, electrolytes, urea, creatinine, fasting blood glucose, lipid profile and liver profile were normal. Erythrocyte Sedimentation Rate (ESR) was 20 mm per first hour and C-reactive protein level was 7 mg/L (normal 0.08–3.1 mg/L). Gadolinium enhanced MRI brain was unremarkable except for mild cerebral atrophy and volume loss compatible with age. She had been previously treated in private clinics by several general practitioners and a neurologist with paracetamol, diclofenac sodium, mefenamic acid, tramadol, flunarizine and sodium valproate without success. She had used these drugs either singly or in varying combinations for at least 2 weeks at a time. As her symptoms continued the diagnosis was reevaluated. Chronic nocturnal headache which would awaken an elderly patient from sleep was considered characteristic of hypnic headache and she was started on indomethacin 25 mg three times a day with a proton pump inhibitor. Improvement in headaches was noted from the first day itself. By day 3, there was absolute response to indomethacin. After continuing treatment for 1 month she was symptom free and the dose was reduced. After a while, the patient had discontinued treatment without medical advice, leading to recurrence of the headache and treatment was re-started. At 1 year after diagnosis she remains headache free on a daily low dose of indomethacin without significant adverse effects.

## Discussion and conclusions

Hypnic (or alarm clock) headache is an uncommon headache disorder. It primarily occurs in age groups over 50 years and is female predominant [1, 2]. It is typically a nocturnal headache, occurring in sleep and waking the patient up, hence the name “alarm clock headache”. It is commonly bilateral but can be unilateral and usually lasts 2–3 h. It is commonly dull or throbbing in character and does not make the patient restless, unlike in Cluster Headache. Most patients engage in some activity after waking up (eating, drinking, showering, reading etc.) [1]. Some patients report improvement with a cup of coffee [3] (which our patient was unwilling to try due to her concern over possible gastric irritation). Sometimes it can be associated with trigeminal autonomic features such as rhinorrhea, tearing and ptosis [1, 2]. Diagnosis is mainly clinical and underlying causes must be excluded. International Classification of Headache Disorders 3^rd^ Edition (ICHD-3)-beta [5] provides diagnostic criteria for hypnic headache. Clinical trial evidence for treatment is lacking and the usual empiric treatment options include indomethacin, lithium, caffeine and melatonin which is structurally similar to caffeine [1–6]. A recent study has suggested that lithium appears to be the most effective treatment option [6]. It was not tried in our patient as her symptoms responded well to the more readily available indomethacin. Indomethacin is noted to be particularly useful if headache is unilateral [1, 2], as in this case. Given its relationship with a variety of headache disorders, indomethacin has been the subject of various studies and reviews. While some headache disorders like hypnic headache, primary stabbing headache, primary cough headache and hemicrania spectrum headaches are responsive to indomethacin, some are resistant. (e.g. Cluster headache). Why indomethacin is better in headache management than other non-steroidal anti-inflammatory drugs remains unclear [7, 8]. A recent case report described improvement of hypnic headache with greater occipital nerve block when all pharmacological options failed [9]. Pathophysiology of hypnic headache is not clearly identified. Currently available evidence suggests involvement of posterior hypothalamus as in Cluster Headache [10]. Our patient fulfilled the ICHD 3-beta criteria for Probable Hypnic Headache. Absence of autonomic features is a required criterion but some patients do report autonomic manifestations [1, 2, 6], as noted in our patient. She did not meet the criteria for related headache disorders like cluster headache and the hemicrania spectrum. Cluster headache usually occurs in younger males, produces excruciating pain with prominent autonomic features and has poor response to indomethacin. Paroxysmal hemicranias do respond to indomethacin but are excruciating and more frequent (more than 5/day as per ICHD-3). Other indomethacin responsive headache disorders like primary cough headache and primary stabbing headache were unlikely in our patient, as the main clinical criteria were not present. Secondary causes of chronic daily headache such as tumours were excluded in our patient with normal MRI imaging. Nocturnal hypertension may cause a similar headache syndrome [11]. Multiple blood pressure measurements were normal in our patient including recordings during episodes of nocturnal headache. The exploding head syndrome is another rare nocturnal disorder seen especially in women over 50 years. However it is rather an auditory sensation than pain and is described as a sudden momentary snap or a pistol shot sound while falling asleep. Our patient had a recurrence of headache after discontinuation of indomethacin, but she has been compliant with medication since then. While most headaches are benign and can be treated easily, some of the rare headache disorders can cause distress to both the patient and the clinician. Difficulties in their diagnosis inevitably leads to extensive investigations and delays in proper treatment. More awareness about the uncommon headache disorders and their management can lead to early initiation of appropriate treatment and significant cost savings.

## Abbreviations

ICHD-3 beta: International Classification of Headache Disorders third edition beta
MRI: Magnetic Resonance Imaging
